# Mechanical Properties of Additively Manufactured Polymeric Materials—PLA and PETG—For Biomechanical Applications

**DOI:** 10.3390/polym16131868

**Published:** 2024-06-29

**Authors:** Rui F. Martins, Ricardo Branco, Miguel Martins, Wojciech Macek, Zbigniew Marciniak, Rui Silva, Daniela Trindade, Carla Moura, Margarida Franco, Cândida Malça

**Affiliations:** 1 UNIDEMI, Department of Mechanical and Industrial Engineering, Nova School of Science and Technology, Universidade NOVA de Lisboa, Campus de Caparica, 2829-516 Caparica, Portugal; mco.martins@campus.fct.unl.pt; 2 Laboratório Associado de Sistemas Inteligentes, LASI, 4800-058 Guimarães, Portugal; 3 Department of Mechanical Engineering, CEMMPRE, ARISE, University of Coimbra, Rua Luís Reis Santos, Pinhal de Marrocos, 3030-788 Coimbra, Portugal; 4 Faculty of Mechanical Engineering and Ship Technology, Gdańsk University of Technology, Narutowicza 11/12, 80-233 Gdańsk, Poland; wojciech.macek@pg.edu.pl; 5 Department of Mechanics and Machine Design, Opole University of Technology, Mikołajczyka 5, 45-271 Opole, Poland; z.marciniak@po.edu.pl; 6 Centre for Rapid and Sustainable Product Development, CDRSP, Polytechnic of Leiria, Rua de Portugal, 2430-028 Marinha Grande, Portugal; rui.d.silva@ipleiria.pt (R.S.); daniela.trindade@ipleiria.pt (D.T.); margarida.franco@ipleiria.pt (M.F.); 7 CIPER, Faculdade de Motricidade Humana, Universidade de Lisboa, 1495 Cruz Quebrada Dafundo, 1649-004 Lisbon, Portugal; 8 Applied Research Institute, Polytechnic Institute of Coimbra, Rua da Misericórdia, Lagar dos Cortiços, S. Martinho do Bispo, 3045-093 Coimbra, Portugal; carla.moura@ipc.pt; 9 Research Centre for Natural Resources Environment and Society, CERNAS, Polytechnic Institute of Coimbra, Bencanta, 3045-601 Coimbra, Portugal; 10 Department of Mechanical Engineering, Polytechnic Institute of Coimbra, ISEC, Rua Pedro Nunes, 3030-199 Coimbra, Portugal

**Keywords:** additive manufacturing, material extrusion, polymers, biomechanical, mechanical properties, fatigue resistance

## Abstract

The study presented herein concerns the mechanical properties of two common polymers for potential biomedical applications, PLA and PETG, processed through fused filament fabrication (FFF)—Material Extrusion (ME). For the uniaxial tension tests carried out, two printing orientations—XY (Horizontal, H) and YZ (Vertical, V)—were considered according to the general principles for part positioning, coordinates, and orientation typically used in additive manufacturing (AM). In addition, six specimens were tested for each printing orientation and material, providing insights into mechanical properties such as Tensile Strength, Young’s Modulus, and Ultimate Strain, suggesting the materials’ potential for biomedical applications. The experimental results were then compared with correspondent mechanical properties obtained from the literature for other polymers like ASA, PC, PP, ULTEM 9085, Copolyester, and Nylon. Thereafter, fatigue resistance curves (S-N curves) for PLA and PETG, printed along 45°, were determined at room temperature for a load ratio, R, of 0.2. Scanning electron microscope observations revealed fibre arrangements, compression/adhesion between layers, and fracture zones, shedding light on the failure mechanisms involved in the fatigue crack propagation of such materials and giving design reference values for future applications. In addition, fractographic analyses of the fatigue fracture surfaces were carried out, as well as X-ray Computed Tomography (XCT) and Thermogravimetric (TGA)/Differential Scanning Calorimetric (DSC) tests.

## 1. Introduction

Biomechanics is a multidisciplinary field that applies the principles of classical mechanics to various biological problems, combining engineering mechanics with biology and physiology. Different aspects of biomechanics utilise concepts and methods from applied mechanics, such as statics for analysing the forces in joints and muscles, dynamics for describing motion and forces in sports mechanics, the mechanics of deformable bodies for evaluating the behaviour of biological materials, and fluid mechanics for studying blood and airflow in the body [1].

Additionally, additive manufacturing (AM) is probably the current production process that will revolutionise the industry in this century. It allows for the production of small batches of highly customised components with very complex shapes and minor postprocessing operations that meet each person’s anatomical requirements.

This study investigates the *quasi*-static mechanical properties of two polymers, PLA and PETG, capable of being used in biomechanical applications obtained through AM. In fact, the AM of polymeric materials has expanded into several areas of engineering, including biomechanics, over the last few years. Biomechanical devices are crucial in improving the quality of life of individuals with disabilities, injuries, or physical limitations, with their vast and diverse range of applications from prostheses to surgical tools and implants [2]. However, there are still challenges to be overcome, especially issues related to strength, toughness, and durability, where it is essential to ensure that these materials have an adequate service life under real conditions of use. For example, the hip joint can be subjected to magnitudes of 870% human body weight (BW) [3] or 1000% BW [4] during stumbling or jumping, respectively, and there are also forces of varying amplitudes resulting from the fatigue loading associated with walking at different speeds, running, and jumping, for example. Therefore, whether AM or conventionally manufactured and whether made of polymers [5] or metallic materials [6,7], prosthetic materials must be designed to endure and support such physical demands. In addition, with the advancement of AM technology, prostheses are becoming increasingly advanced and sophisticated, offering a greater functionality and comfort for patients together with an improved design [8].

At the same time, the ASTM International Technical Committee F42 defined AM as a mechanical process of joining materials to make objects from three-dimensional (3D) model data, usually layer upon layer, instead of subtractive manufacturing methodologies [9]. This enables the production of end products, encompassing prototypes for design verification—shape and fit checking, tools, and conceptual components [10] with increased design complexity [11] that could not be easily obtained with a conventional manufacturing process. Additionally, the absence of tooling requirements in AM leads to a reduction in the production ramp-up time and costs, enables the production of small batches that are both feasible and cost-effective, and offers the advantage of quickly modifying designs, optimising products for specific functions, reducing waste, and using few raw materials [11]. Furthermore, it holds the potential for simplified supply chains, shorter lead times, lower inventories, and the ability to customise designs to meet specific requirements [12]. Nevertheless, AM does face certain limitations that restrict its wide-scale application, namely surface finishing, as achieving the desired quality can be challenging without post-processing. Moreover, the build space of AM machines also imposes physical constraints on the component dimensions, thereby limiting the size of the produced parts to small ones [13]. However, the issue of not producing large AM parts is gradually being overcome [14].

In addition, ASTM [9] defined eight different categories to accommodate the existing and future AM machine technologies, such as Material Extrusion (ME), Material Jetting, Binder Jetting, Sheet Lamination, Vat Photopolymerisation, Powder Bed Fusion, Directed Energy Deposition, and Cold Spraying. In this study, the specimens for mechanical experimental tests were obtained using ME, which utilises a filament of thermoplastic material as the feedstock. This thermoplastic filament is fed into a temperature-controlled extrusion head; after that, the filament is heated until it reaches a semi-liquid state, and it is then extruded and deposited in ultra-thin layers onto a build platform that solidifies quickly upon deposition, creating a solid part. One of the critical advantages of ME is its versatility in material selection, which has various types of thermoplastics at its disposal, offering a wide range of mechanical and thermal properties. Additionally, there are many variables that influence the resulting mechanical properties, including filament manufacturing, process parameters such as layer thickness, printing speed, printing head temperature, build plate temperature, build orientation, raster angle, 3D-printing equipment, ageing, and post-process treatments, and mechanical testing procedures [15].

**Figure 1:**
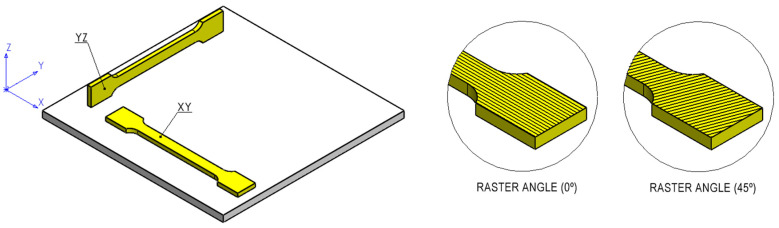
Printing orientations. Alternating raster angle: 0° (*quasi*-static tests, XY and YZ orientations) and 45°/−45° (fatigue tests, XY orientation).

During this investigation, a raster angle equal to 0° and two printing orientations—XY (Horizontal, H) and YZ (Vertical, V)—were considered according to the general principles for part positioning for *quasi*-static tensile specimens [16], where the first characteristic dimension is the length of the specimen and the second characteristic dimension is the width of the specimen [15] (Figure 1). For the fatigue tests, an alternating raster angle equal to 45°/−45° and an XY orientation was used (Figure 1).

## 2. Materials and Methods

### 2.1. Uniaxial Tensile Tests and Fatigue Tests

The experimental test specimens were 3D printed using an Anycubic Kobra printer, Shenzhen, China, with a 100% infill density, a layer height of 0.25 mm, a shell thickness of 2 mm, a print speed of 50 mm/s, a filament diameter of 1.75 mm, and a nozzle diameter of 0.4 mm. The printing temperature typically ranged from 215 °C to 220 °C, and the bed temperature was 70 °C.

Commercial filaments of PLA and PETG from Stratasys [17] were used for the fabrication of all the specimens tested under the *quasi*-static uniaxial load and the fatigue loading.

**Figure 2:**
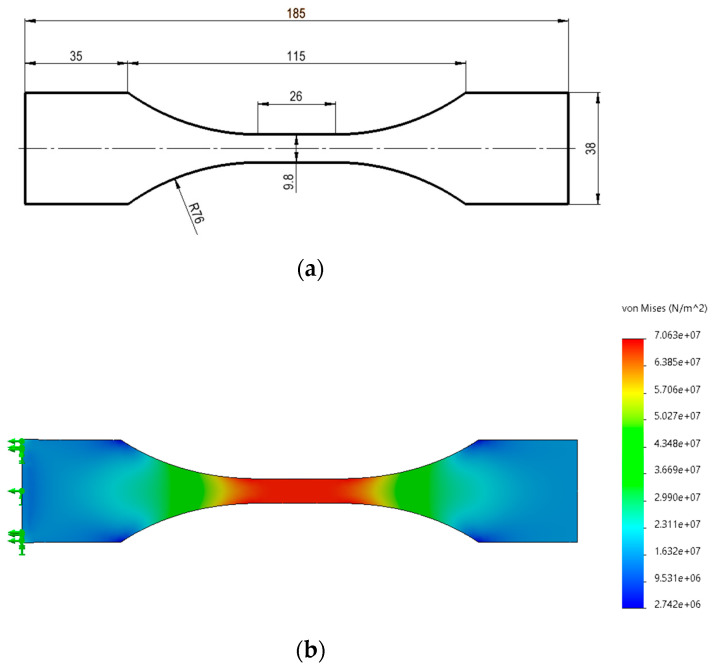
(**a**) Specimen dimensions [mm] and (**b**) uniaxial tensile test simulation, linear elastic analysis, and von Mises stress distribution [Pa].

The geometry of the specimens (Figure 2) used for the *quasi*-static and fatigue tests were customised considering the geometries and recommendations outlined in the standards ASTM D638-14 [18] and ASTM E466-96 [19]. Moreover, a finite element analysis was carried out to verify the stress distribution on the specimen (Figure 2) and assess the assumed geometric variables’ adequacy. Issues such as the grip cross-sectional area in relation to the test section area, the radius of the blending fillets, the ratio of specimen test section width to thickness, and the test section length in relation to the test section width of the specimen were considered in the definition of the specimens’ geometry. As expected, structural analysis (Figure 2) showed the maximum stress values in the test section area.

The *quasi*-static tensile tests were carried out with a uniaxial testing machine, Instron 5544, with a maximum load capacity of 2 kN. For the uniaxial tension tests carried out, two printing orientations—XY (Horizontal, H) and YZ (Vertical, V)—were considered (Figure 1). In addition, six specimens were tested for each printing orientation and material, providing insights into mechanical properties—average and standard deviation—such as Tensile Strength (UTS), Young’s Modulus, and Ultimate Strain. The testing speed was defined as 1 mm/min, according to ASTM D638 [18], to produce the specimens’ rupture in 3 to 7 min.

PLA and PETG were chosen for the fatigue testing due to their wide usage in various applications and low cost. High-cycle fatigue tests were performed with an MTS servohydraulic testing machine with a load frame capacity of 100 kN, a load ratio, R, equal to 0.2, and a load frequency of 7 Hz. Thereafter, fatigue resistance curves (S-N curves) for PLA and PETG, printed along alternating 45°/−45° angles, were determined at room temperature. To carry out the fatigue tests effectively, at least three different stress levels were established, including 60%, 45%, and 30% of the tensile strength (UTS) for the two materials, and each stress level underwent three repeated tests (3 specimens per stress level) to ensure statistical significance and robust data collection.

### 2.2. TGA/DSC Samples

The test was carried out with a Simultaneous Thermal Analyzer (STA 6000 Perkin Elmer, Waltham, MA, USA) with nitrogen as the purge gas at a flow rate of 20 mL/min. The heating was from 30 °C to 600 °C with a heating rate of 10 °C/min. The PETG’s sample weights were around 6.88 ± 0.48 mg and PLA’s sample weights were around 6.03 ± 0.76 mg. The Thermogravimetric Analysis (TGA) and the Differential Scanning Calorimetric (DSC) were evaluated. Three samples of each material were analysed.

### 2.3. X-ray Computed Tomography (XCT)

The XCT tests were carried out with a SkyScan1174v2 scanner, Bruker (Billerica, MA, USA). The scan parameters selected were a source voltage of 50 kV, source current of 800 µA, image pixel size of 30.11 µm, exposure of 8000 ms, and rotation step equal to 0.900 deg.

### 2.4. SEM Images and Fractographic Analyses

The SEM images of the fractured surfaces were taken using a Hitachi High-Tech Model SU3800 (Hitachi, Tokyo, Japan) under a low-vacuum mode for non-conductive materials at 30 Pa of pressure and with an accelerating voltage of 20 kV. Using this mode, images were captured at different magnifications.

In addition, a fractographic examination of the fracture surfaces was performed according to the entire fracture surface method [20]. They were subsequently measured using the Sensofar S-Neox optical profilometer (Sensofar, Barcelona, Spain) with the focus variation method (FVM) [21]. The fracture surfaces were observed under 10× magnification and stitched with a 7 × 3 grid to map the entire fracture area, with a pixel size of 1.38 μm/pixel. Sensofar (.plux) source files were transferred into the surface texture analysis software MountainsMap (version 7.4, Digital Surf, Besançon, France) and resampled into height maps at a resolution automatically set by the software. The main fractographic features are reflected by the surface topography parameters [22].

## 3. Results and Discussion

### 3.1. Uniaxial Tensile Quasi-Static Tests

**Figure 3:**
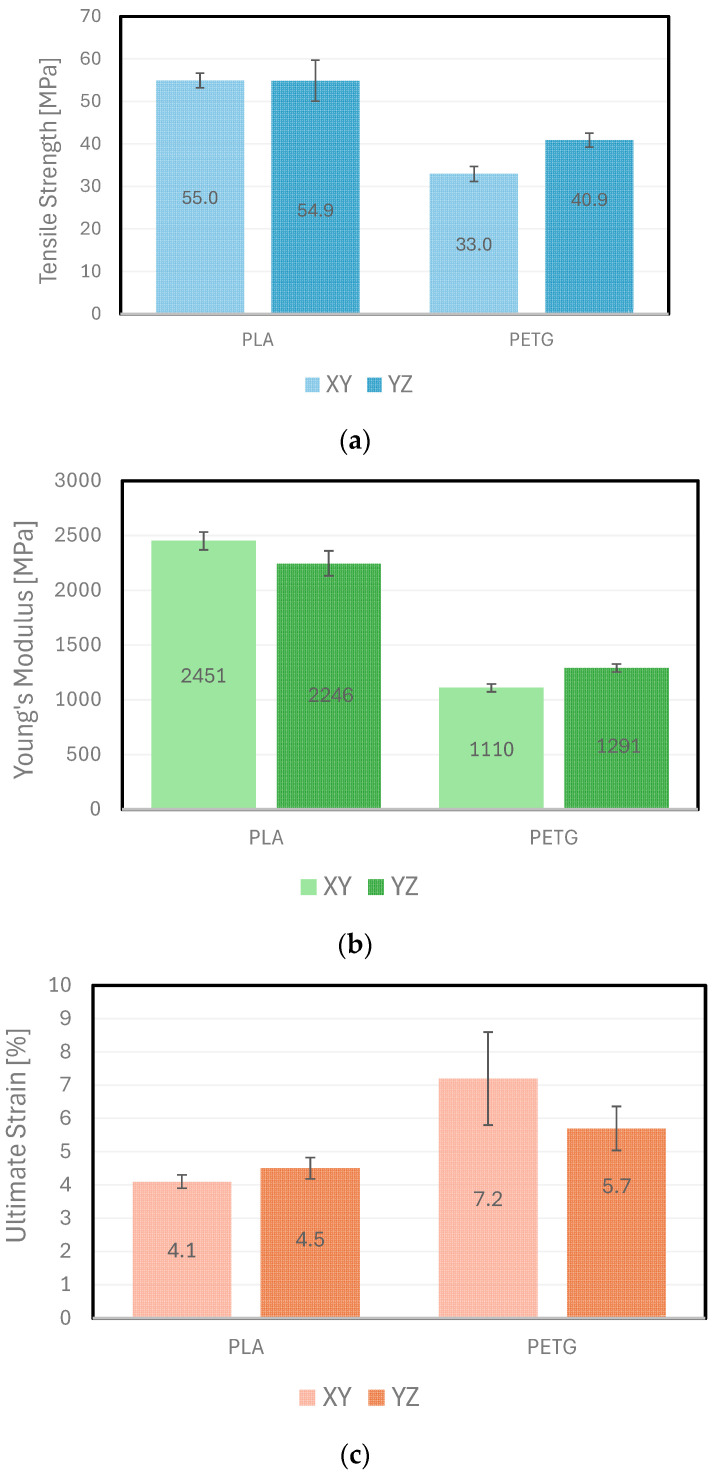
Comparison between mechanical properties of PLA and PETG under tensile testing: (**a**) Tensile Strength; (**b**) Young’s Modulus; and (**c**) Ultimate Strain.

Table 1 and Figure 3 present the values of the Tensile Strength, Young’s Modulus, and Ultimate Strain of the two materials tested, PLA and PETG, grouped by the printing orientation (XY or YZ).

Considering the results of the uniaxial tensile tests, the following conclusions may be drawn:

- For the XY and YZ printing directions, the Tensile Strength and Young’s Modulus (average values) obtained for PLA (55 MPa|2350 MPa) were higher than the corresponding values obtained for PETG (37 MPa|1200 MPa) by approximately 48% and 96%, respectively;
- PLA may, therefore, be used for components under induced stresses up to 55 MPa and when greater rigidity is required, as PLA revealed higher Young’s Modulus values (average value: 2300 MPa);
- PETG may be used for components under induced stresses up to 33 MPa and when higher deflections are allowed, since PETG revealed lower Young’s Modulus values (average value: 1200 MPa);
- The average ultimate strain values obtained for PLA and PETG, either printed along XY or YZ, were around 4.3% and 6.5%, respectively. Therefore, PETG showed more ductile behaviour than PLA;
- The experimental results obtained for PETG and PLA were in the same order of magnitude of the manufacturer’s specifications [17], with deviations of −18%, and +22%, respectively. However, it is important to stress that the manufacturer’s values are typically provided for the XZ orientation, while the tests were conducted in the XY and the YZ planes;
- Moreover, comparing the mechanical properties of PLA and PETG with other polymeric materials, the tensile strength value of ULTEM^®^ 9085 was the highest of the common AM polymeric materials tested and was about 70 MPa [23]; a second group of materials—PC and PLA—demonstrated tensile strength values around 55 MPa [24,25]; a third group included ASA, PP, ABS, and PETG, with tensile strength values of approximately 35 MPa [24,25]; and a fourth group of materials included Nylon and Copolyester with tensile strength values of about 20 MPa [25]. Therefore, the experimental tensile strength results (Table 1 and Figure 3) compare well with the published data [23,24,25].
- The differences between the Young’s Moduli for the different polymeric materials referred to above were around 1500 MPa [24,25]. In fact, PLA revealed the highest Young’s Modulus values (around 2600 MPa [24,25], which were similar to the average value of 2350 MPa presented in Table 1 and Figure 3, and ASA/Nylon/Copolyester revealed the lowest (500 MPa) [25]. PETG Young’s Modulus was about 1500 MPa [25], which also compares reasonably well with the experimental data (1200 MPa, Table 1 and Figure 3).

In addition, statistical analyses between the printing directions, XY and YZ, were evaluated on GraphPad Prism 9 software with a two-way ANOVA with a Fisher’s Least Significant Difference test, where statistically significant differences are represented by *p* < 0.01 and *p* < 0.001. Comparing the results obtained, PETG showed statistically significant differences across all the parameters analysed, namely Tensile Strength, Young’s Modulus, and Ultimate Strain. Specifically, the vertically printed specimens (YZ) exhibited a higher Tensile Strength (*p* < 0.001) and Young’s Modulus (*p* < 0.01) compared to the horizontally printed ones (XY). Conversely, for the Ultimate Strain, the horizontally printed PETG specimens (XY) showed higher values (*p* < 0.01).

In the case of PLA, statistically significant differences were only observed in the Young’s Modulus (*p* < 0.001), with the horizontally printed specimens (XY) demonstrating superior values.

This discrepancy was likely due to variations in the interlayer temperature and hatch distance, which can affect the adhesion between layers, the existence of voids, and the consistency of the deposited filament’s geometry, leading to differences in mechanical performance [26].

### 3.2. Uniaxial Tensile Fatigue Tests

PLA and PETG were chosen for the uniaxial tensile fatigue testing under load control due to their wide usage in various applications and low cost. To carry out these tests effectively, at least three different stress levels were established, including 60%, 45%, and 30% of the ultimate tensile strengths of those two materials obtained from the *quasi*-static uniaxial tensile tests. Each stress level was intended to undergo three repeated tests. The thickness of each test specimen was constant. However, some specimens were approximately 5 mm thick, while others were about 7 mm thick. This explains the variation in the forces applied within each stress level tested (Table 2 and Table 3).

**Figure 4:**
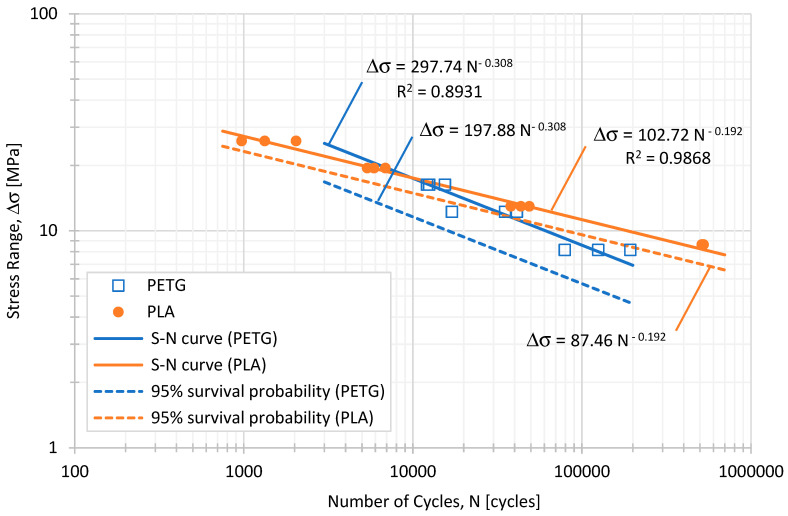
Fatigue strength of PLA and PETG. Load ratio, R, equal to 0.2.

For PETG and the above-chosen test levels, maximum stress values, σ_max_, of approximately 20.4 MPa, 15.3 MPa, and 10.2 MPa were applied, respectively (Table 2); when considering PLA, the maximum stress values applied were equal to 32.4 MPa, 24.3 MPa, and 16.2 MPa (Table 3). Cyclic loading was applied during the fatigue tests, oscillating between the maximum stress and the minimum stress, σ_min_, set at 20% of the maximum stress applied (load ratio, R = 0.2), see Table 2 and Table 3. The fatigue life results obtained for both materials are shown in Figure 4.

As shown in Figure 4, PLA demonstrates a higher fatigue strength, withstanding higher stresses than PETG for the same fatigue life. Additionally, the slope of the PETG curve indicates a more rapid decrease in the fatigue life as the stress levels decreasd compared to PLA. In addition to the standard S-N curve represented by the full lines, a fatigue design curve was drawn for a 95% survival probability, determined from the mean curve with a confidence level of 95%. In the case of PLA, the scatter associated with the experimental data is smaller than that for PETG, which explains why the fatigue design curve is closer to the S-N curve.

The results found in scientific databases regarding the high-cycle fatigue (HCF) of PLA and PETG under uniaxial tensile loads are extremely scarce. Some studies combine PLA with flax fibres [27] or PCL [28]; others include PLA coating with an AM60 magnesium alloy [29]. No HCF results were found for PETG. Algarni [30] studied the PLA fatigue behaviour for specimens with large, medium, and sharp notches, which differs from the results obtained in this investigation. Only in the paper by Azadi et al. [31] was it possible to find some HCF results for PLA obtained under fully reversed stress-controlled bending loading (R = −1). Moreover, the layer thickness was 0.15 mm, the infill percentage of the parts was 50%, and the specimens were cylindrical. Nevertheless, the authors found a Basquin’s exponent equal to −0.288, which compares with the value determined experimentally in this study (−0.192). Additionally, Afrose et al. [32] observed that, under low-cycle fatigue loading, the PLA specimens built in 45°-orientations achieved the highest fatigue life compared to those PLA specimens built in the X- and Y-orientations. From the results presented by the authors [32], it was possible to adjust a power law with an exponent equal to −0.165, which compares reasonably well with −0.192.

### 3.3. TGA/DSC Results

**Figure 5:**
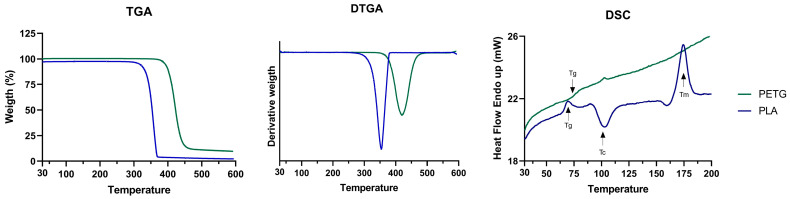
TGA, DTGA, and DSC results for PETG and PLA.

The derivative of the TGA (DTGA) curves of PLA (Figure 5) showed thermal decomposition (T_d_) at 357.57 ± 0.69 °C, and by TGA, it was possible to obtain a residual waste of 1.27 ± 0.9%, meaning that PLA almost totally decomposed. Since PLA is a semi-crystalline thermoplastic, the DSC curves showed a glass transition temperature (T_g_) of 69.25 ± 0.93 °C, an exotherm peak associated with a cold crystallization temperature (T_c_) of 102.74 ± 0.46 °C, and a melting temperature (T_m_) of 174.71 ± 1.35 °C (Figure 5).

On the DTGA curve of PETG, the T_d_ was 419.27 ± 1.86 °C, and was accompanied by a residual waste of 11.12 ± 1.46%, meaning that the PETG did not totally decompose. Since PETG is an amorphous material, it only presented a T_g_ of 73.46 ± 0.78 °C.

The printing process can significantly influence the material properties, greatly impacting the mechanical stability of the polymers. However, the observed values aligned with the existing literature for PLA and PETG [33,34,35], and with Ronca et al., who demonstrated that thermal properties remain unaffected by the printing process [36].

### 3.4. XCT Results

**Figure 6:**
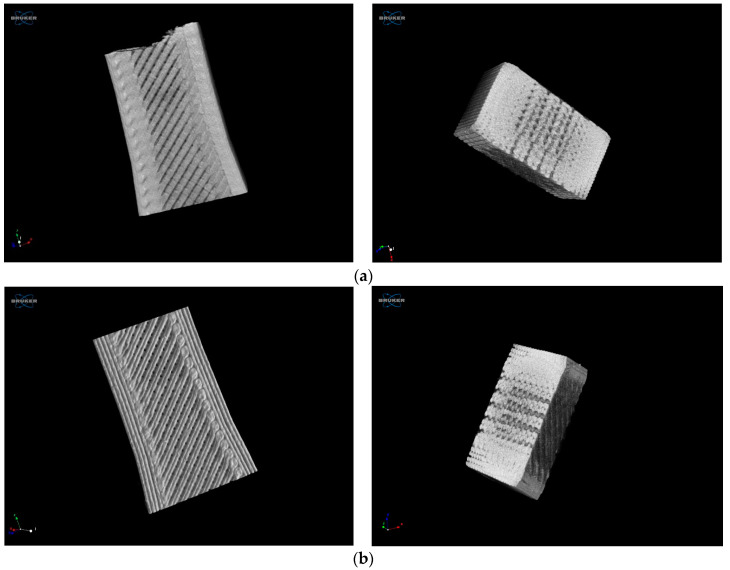
XCT results: (**a**) PLA and and (**b**) PETG.

The morphometry results revealed a total porosity of 9.3% for the specimens made of PLA and 12% for those made of PETG (Figure 6). These values were obtained for 350 layers, with lower and upper grey thresholds of 70 and 255, respectively, and considering a total Volume-of-Interest (VOI) volume of 2.1667 × 10^11^ μm^3^ for all samples observed.

Although the infill percentage was 100%, the XCT tests measured total porosity values of 9.3% (PLA) and 12% (PETG). These indicate the proportion of void space within the material, which can significantly impact its mechanical properties and performance. In fact, a high porosity typically leads to a reduced strength, stiffness, fatigue strength, and fracture toughness. Therefore, these porosity values should be considered when interpreting the mechanical testing results, as they could explain the variations in performance between samples or compared to theoretical predictions, as stated by Garcias et al. [37], where a penalizing fatigue coefficient was suggested to be applied to porosity/defects introduced by additive manufacturing.

### 3.5. SEM and Fractography Results

**Figure 7:**
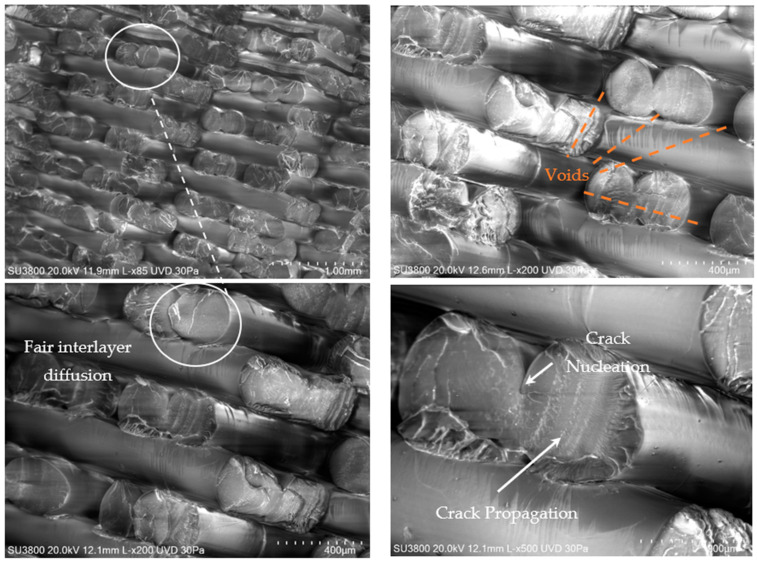
Images of scanning electron micrographs after fatigue testing of PLA.

**Figure 8:**
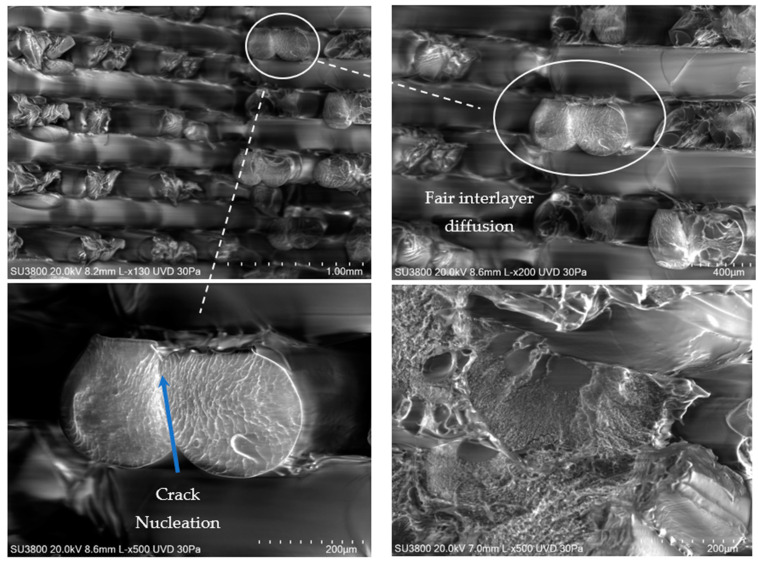
Images of scanning electron micrographs after fatigue testing of PETG.

Figure 7 and Figure 8 show SEM images of the fractured surfaces caused by fatigue loading. Fair interlayer diffusion and a good diffusion between the pairs of deposited material for each layer height can be seen. This arrangement of pairs of the deposited material also confirmed visible air voids already detected by the XCT results. From the images, perceptible nucleation points and fatigue striation due to fatigue crack propagation can also be seen. The arrangement of the material and failure mechanisms were similar in PLA and PETG.

Air voids were caused by the manufacturing printing strategy and hatch distance resulting from the deposition of contiguous lines. As stated in the previous Section 3.4, these voids can significantly impact the material properties in several ways. In fact, air voids reduce the effective cross-sectional area of the material, leading to a decreased load-bearing capacity. This results in lower tensile and compressive strengths and a reduced stiffness. Moreover, the presence of air voids can act as stress raisers. Therefore, under cyclic loading (fatigue), these stress concentrators can initiate cracks, significantly reducing the fatigue resistance of the material. This makes the material less suitable for applications where it will be subjected to repetitive loading. Moreover, voids can also reduce the material’s fracture toughness, making it more susceptible to unstable and non-predicted catastrophic failure. Nevertheless, while materials with a high porosity and significant air voids may not be ideal for structural applications that demand a high strength and durability, they can still find utility in specific biomechanical applications and situations where lightweight materials are advantageous, and the mechanical load is less demanding. Comparing these findings with other studies [37], it is evident that the presence and distribution of air voids/defects and nucleation points are critical factors influencing the performance of materials. Moreover, studies have also shown that reducing the porosity through optimized processing conditions and enhancing the strength of the fibre bond can significantly improve the mechanical properties of these materials [38,39]. Hence, the SEM results indicate that understanding and controlling the additive process parameters are crucial for optimizing the material properties for specific applications.

**Figure 9:**
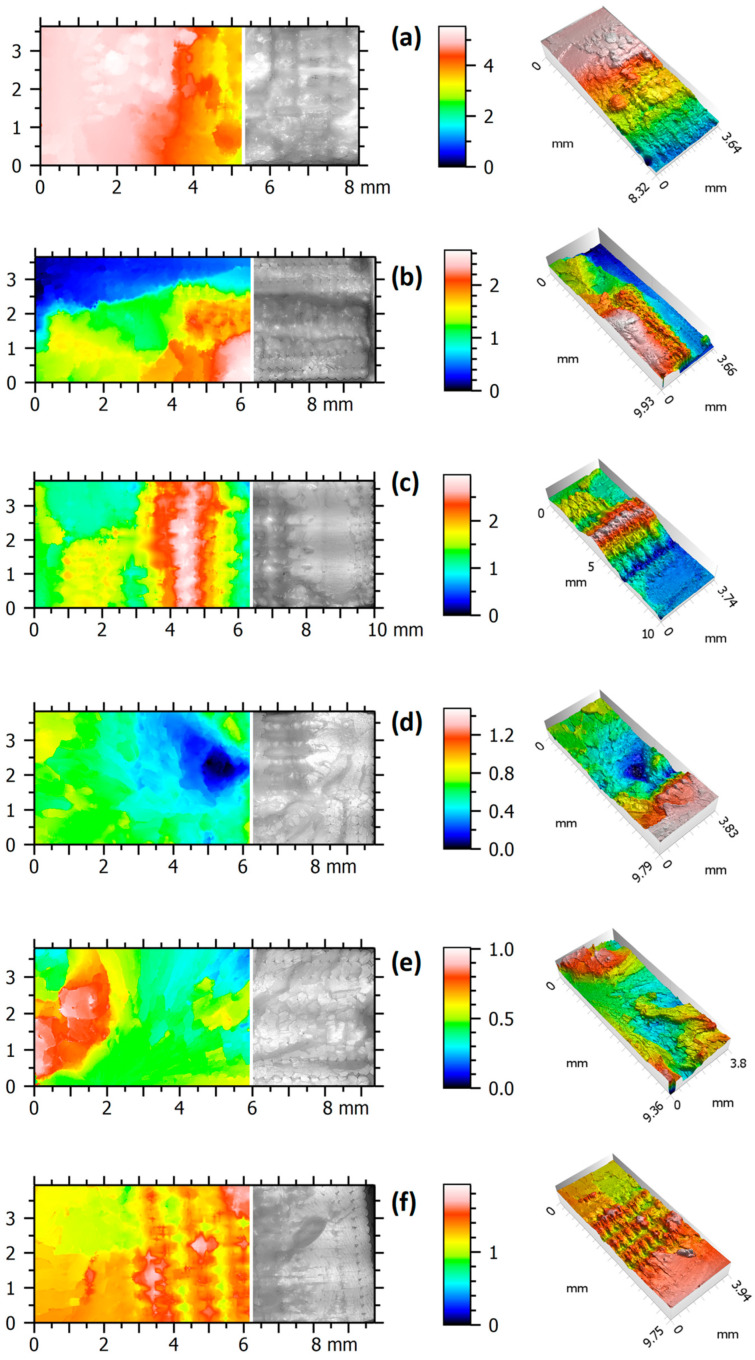
Surface and 3D views of specimens tested under monotonic and fatigue loading: (**a**) PETG H (monotonic); (**b**) PETG V (monotonic); (**c**) PETG, specimen 26 (fatigue); (**d**) PLA H (monotonic); (**e**) PLA V (monotonic); and (**f**) PLA. specimen 14 (fatigue).

Regarding the 3D fractography results, pseudo-colour views of the surface and 3D views, respectively, ordered from left to right, for representative specimens are presented in Figure 9. On the scales, it can be observed that the largest differences in surface height occurred for the specimen PETG H (see Figure 9a) and the smallest for the specimen PLA V (see Figure 9e).

**Figure 10:**
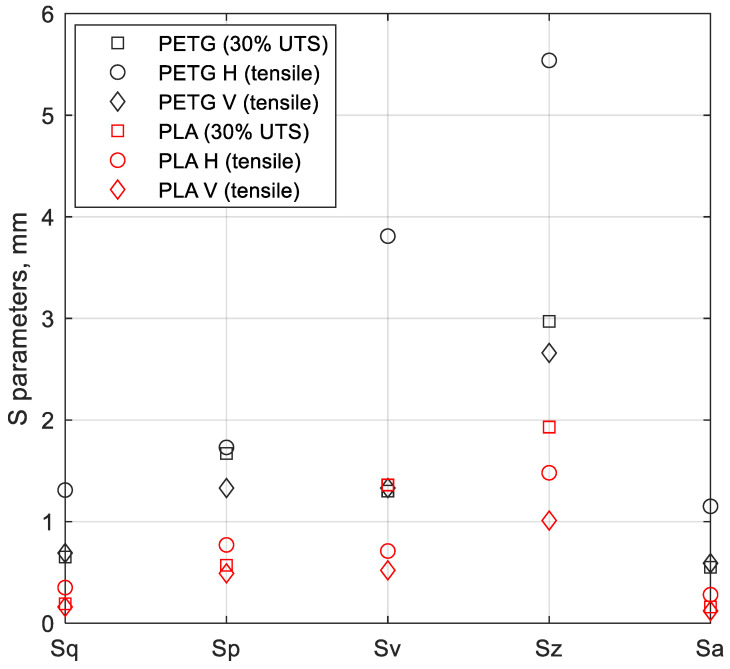
The dependence of the areal surface topography parameters from the printing direction and the loading type for PETG and PLA.

The dependence of the areal surface topography parameters from the manufacturing method is presented in Figure 10. Areal surface topography parameters are usually identified by an initial capital letter S. In this study, the root-mean-square height (Sq), maximum peak height (Sp), maximum valley depth (Sv), maximum height of the surface (Sz), and arithmetic mean height (Sa) were considered. Figure 9 shows examples of specimens subjected to fracture surfaces topography analysis, which includes different materials, different printing directions, and different types of applied loading.

For all specimens and all surface topography parameters obtained in this study, the PETG specimens had higher values (black markers) when compared with their counterparts made of PLA. For each of the analysed specimens, the surface topography parameters were the highest for H (after tensile test) and the lowest for V (after tensile test), respectively. The unique exception was related to the Sv and Sz values for the PLA specimen tested at 30% of its UTS (red circle marker). This was due to the large peak height found in the fracture surface of this specimen to which these parameters are sensitive.

## 4. Conclusions

As the summary and introduction state, this study was dedicated to determining the mechanical (tensile and fatigue) and physical (TGA, DTGA, DSC, XCT, and SEM) properties of two widely available, lightweight, and cost-effective materials, PLA and PETG. These materials hold promise for future use in biomechanical applications that are not subjected to high loads, such as prostheses, surgical tools, and implants manufactured through additive manufacturing (material extrusion).

PLA and PETG have been widely used in additive manufacturing for several years to create specimens and components. However, the information about these materials, particularly regarding fatigue, is very scarce. Therefore, the study successfully achieved its objectives, leading to the following key conclusions:

- The uniaxial tensile tests yielded valuable data, including critical parameters, such as the maximum Tensile Strength, Young’s Modulus, and Ultimate Strain, for two printing orientations (XY and YZ). These results suggest that all materials studied exhibited promising mechanical characteristics suitable for general biomechanical use;
- For the XY and YZ printing directions, the Tensile Strength and Young’s Modulus (average values) obtained for PLA (55 MPa|2350 MPa) were higher than the corresponding values obtained for PETG (37 MPa|1200 MPa) by approximately 48% and 96%, respectively;
- PLA may, therefore, be used for components under induced stresses or up to 55 MPa and when greater rigidity is required, as PLA revealed higher Young’s Modulus values (around 2300 MPa);
- PETG may be used for components under induced stresses up to 33 MPa and when higher deflections are allowed, since PETG revealed lower Young’s Modulus values (around 1200 MPa);
- The average ultimate strain values obtained for PLA and PETG, either printed along XY or YZ, were around 4.3% and 6.5%, respectively. Therefore, PETG showed more ductile behaviour than PLA;
- In addition, uniaxial tensile fatigue testing (R = 0.2) under load control was conducted on the PLA and PETG materials at three stress levels, with the results depicted in the form of stress–life (S-N) curves. These curves may point to the suitability of these materials for continuous and durable use in biomechanical applications depending on the spectrum loading applied;
- XCT, SEM, and fractography analyses of the tested materials uncovered essential features, including fibre arrangements, compression issues related to interlayer adhesion and hatch distance, voids within fibre connections, and failure mechanisms;
- The surface topography parameters for the PETG specimens had higher values than PLA, irrespective of the printing direction or type of loading.

## Figures and Tables

**Table 1:** Mechanical properties of PLA and PETG under tensile testing (average ± standard deviation).

XY			
	Tensile Strength [MPa]	Young’s Modulus [MPa]	Ultimate Strain [%]
PLA	54.9 ± 1.73	2451.4 ± 81.12	4.1 ± 0.17
PETG	32.9 ± 1.77	1109.6 ± 36.27	7.3 ± 1.38
YZ			
	Tensile Strength [MPa]	Young’s Modulus [MPa]	Ultimate Strain [%]
PLA	54.9 ± 4.82	2245.7 ± 114.80	4.5 ± 0.32
PETG	40.9 ± 1.64	1290.7 ± 36.02	5.7 ± 0.66

**Table 2:** Fatigue results obtained for specimens made of PETG. Load ratio, R, equal to 0.2. Frequency, f = 7 Hz. Maximum force (F_max_), minimum force (F_min_), amplitude force (F_a_), and medium force (F_med_).

Spec. #	Thickness [mm]	Stress Level	σ_max_ [MPa]	σ_min_ [MPa]	Stress Range, ∆σ [MPa]	F_max_ [N]	F_min_ [N]	F_a_ [N]	F_med_ [N]	N_cycles_
1	5.06	60% UTS	20.4	4.08	16.31	1012.2	202.4	404.9	607.3	12,132
2	5.04					1008.3	201.7	403.3	604.9	12,496
3	4.98					996.8	199.4	398.7	598.1	15,483
4	7.10	45% UTS	15.3	3.06	12.23	1064.6	212.9	425.8	638.7	17,040
5	5.06					758.5	151.7	303.4	455.1	41,250
6	5.06					758.3	151.7	303.3	454.9	35,140
7	7.13	30% UTS	10.2	2.04	8.16	712.8	142.6	285.1	427.7	79,271
8	5.09					509.1	101.8	203.6	305.5	124,889
9	5.14					514.1	102.8	205.7	308.5	193,323

**Table 3:** Fatigue results obtained for specimens made of PLA. Load ratio, R, equal to 0.2. Frequency, f = 7 Hz. Maximum force (F_max_), minimum force (F_min_), amplitude force (F_a_), and medium force (F_med_).

Spec. #	Thickness [mm]	Stress Level	σ_max_ [MPa]	σ_min_ [MPa]	Stress Range, ∆σ [MPa]	F_max_ [N]	F_min_ [N]	F_a_ [N]	F_med_ [N]	N_cycles_
10	7.10	60% UTS	32.4	6.48	25.92	2255.8	451.2	902.3	1353.5	2040
11	5.10					1619.9	323.9	647.9	971.9	1331
12	5.13					1627.7	325.5	651.1	976.6	972
13	5.08	45% UTS	24.3	4.86	19.44	1209.2	241.8	483.7	725.5	6880
14	5.13					1221.5	244.3	488.6	732.9	5900
15	5.15					1225.6	245.1	490.3	735.4	5380
16	5.09	30% UTS	16.2	3.24	12.96	807.6	161.5	323.1	484.6	43,600
17	5.13					814.4	162.9	325.8	488.7	48,893
18	5.18					821.9	164.4	328.8	493.2	37,970
19	5.16	20% UTS	10.8	2.16	8.64	546.6	109.3	218.7	327.9	525,170

## Data Availability

The original contributions presented in the study are included in the article, further inquiries can be directed to the corresponding authors.

## Abbreviations

ASA	Acrylonitrile Styrene Acrylate
AM	Additive Manufacturing
FFF	Fused Filament Fabrication
HCF	High-cycle Fatigue
ME	Material Extrusion
PC	Polycarbonate
PETG	Polyethylene Terephthalate Glycol
PLA	Polylactic Acid
PP	Polypropylene
VOI	Volume-of-Interest
